# Metagenomic profiling of blood-associated microbial DNA signatures in leukemia-associated febrile neutropenia

**DOI:** 10.3389/fmicb.2026.1917043

**Published:** 2026-08-28

**Authors:** Ming Zhang, Lan He, Huaping Xin, Tao Huang, Feijie Wang, Li Wang

**Affiliations:** 1 Department of Statistics, The People’s Hospital of Yichun City, Yichun, Jiangxi, China; 2 First Clinical Medical College of Gannan Medical University, Ganzhou, Jiangxi, China; 3 Department of Information, First Affiliated Hospital of Gannan Medical University, Ganzhou, Jiangxi, China

**Keywords:** antimicrobial resistance genes, blood metagenomics, febrile neutropenia, metagenome-assembled genomes, shotgun metagenomic sequencing

## Abstract

Febrile neutropenia (FN) is a life-threatening complication of chemotherapy, but the low microbial biomass of blood makes shotgun metagenomic profiles highly sensitive to technical background. We reanalyzed 47 publicly available patient sequencing runs representing 43 unique patient-timepoint samples from 19 SRA-labeled patients, together with 23 no-template-control (NTC) runs spanning 21 sequencing batches. To distinguish reference-catalogue content from progressively stronger evidence of patient-associated signal, we applied batch-matched NTC correction together with nested abundance thresholds and a feature-specific global NTC envelope. CheckM2 evaluated 1,013 bins; 13 met completeness ≥50% and contamination <10%, and dereplication yielded 11 draft MAG representatives. Ten representatives showed positive patient-to-control abundance excess, but only four showed recurrent support above both threefold matched-control abundance and the global NTC envelope. Functional annotations were therefore interpreted as reference-genome homologs rather than evidence of expression, phenotype, viability or bloodstream origin. Matched-control correction retained 19 read-level ARG types, but only seven subjects contributed complete longitudinal ARG-profile contrasts, limiting reliable temporal inference. The resulting run-resolved, nested evidence framework identified a subset of microbial DNA and ARG signals that remained detectable under increasingly stringent control criteria while distinguishing them from catalogue-level or background-sensitive signals. These findings support cautious reporting of patient-enriched microbial DNA and ARG signals rather than inference of a resident blood microbiome or clinical resistance phenotype.

## 1 Introduction

Febrile neutropenia (FN) is among the most serious complications of myelosuppressive chemotherapy in patients with hematological malignancies. Profound immunosuppression renders these patients vulnerable to bloodstream infection and rapid clinical deterioration, making timely pathogen identification a clinical priority (Freifeld et al., 2011; Klastersky et al., 2016). Blood culture remains the standard diagnostic method, but its yield is constrained by dependence on microbial growth and by antimicrobial exposure before sampling (Fenollar and Raoult, 2007; Kristóf and Pongrácz, 2016). Shotgun metagenomic sequencing detects microbial nucleic acids without cultivation and can therefore broaden characterization of blood-derived microbial DNA profiles, although sequence detection alone does not establish viable infection (Blauwkamp et al., 2019; Chiu and Miller, 2019).

Blood-associated microbial DNA signals can also contain genes annotated as antimicrobial-resistance, virulence-associated or mobile-element homologs. These annotations may generate clinically relevant hypotheses, but ARG detection does not establish a resistance phenotype, virulence-factor homology does not establish pathogenicity, and MGE-associated genes do not demonstrate active horizontal transfer (Baquero, 2021; Murray et al., 2022; Partridge et al., 2018). Genome-resolved metagenomics can place such homologs within reconstructed draft genomes (Bowers et al., 2017; Quince et al., 2017), but interpretation must account for incomplete genome recovery and the contamination sensitivity of low-biomass blood sequencing.

Longitudinal sampling may help distinguish fever-phase-associated changes from persistent background signals, but incomplete timepoint coverage, antimicrobial exposure and small paired sample sizes can limit inference. External datasets can provide useful contextual comparison only when sample identities, processing methods and controls are sufficiently documented. When run roles cannot be verified, external analyses should be treated as exploratory rather than as independent validation.

In this study, we reanalyzed longitudinal blood shotgun metagenomes from PRJNA674642 with explicit batch-matched NTC integration. Although the influence of background contamination on low-biomass sequencing is well established (Salter et al., 2014; Eisenhofer et al., 2019), its quantitative impact on taxonomic, resistome and genome-resolved signals in this dataset had not been systematically evaluated. We therefore applied a run-resolved, nested evidence framework combining continuous batch-matched background correction, ratio-based sensitivity thresholds and a feature-specific global NTC envelope. This framework separated the full reference catalogue from progressively stronger evidence of patient-associated enrichment and allowed the robustness of taxonomic, resistome and genome-resolved signals to be evaluated across increasingly stringent control criteria. Kraken2-derived read-level taxonomic assignments were analyzed separately from geNomad-based assembled viral-contig screening and GTDB-Tk classification of bacterial and archaeal MAGs. SRP016572 was examined only as exploratory external context because unresolved run identities in the public archive precluded its use as an independent validation cohort.

## 2 Materials and methods

### 2.1 Study design and public datasets

The discovery dataset was obtained from NCBI BioProject PRJNA674642 and the associated source study (Vijayvargiya et al., 2022). The source study enrolled 20 adults with acute myeloid or acute lymphoblastic leukemia and reported 47 biological blood samples: 11 pre-fever samples (T1), 20 samples collected within 24 h of fever onset (T2), and 16 samples collected 5–7 d after fever onset (T3). The public SRA deposit contained 47 patient sequencing runs representing 43 unique patient–timepoint identifiers from 19 SRA-labeled patients (unique public samples: T1 = 9, T2 = 19 and T3 = 15), together with 23 NTC runs spanning 21 sequencing batches. Four patient sample identifiers (5.2, 6.1, 6.2 and 6.3) were represented by duplicate technical runs and were not counted as independent biological observations. Clinical subject 1 was not identifiable in the public SRA. The correspondence of SRA labels patient2–patient20 to clinical subjects 2–20 was treated as probable rather than explicitly documented. Source-labeled cohort characteristics and patient-level clinical fields are provided in Supplementary Tables S1, S2.

### 2.2 Source-study specimen processing and negative controls

All wet-laboratory procedures were performed by the investigators of the source study and are summarized here from the original article and supplementary methods. Whole blood was collected in EDTA tubes and processed with the iDTECT Dx Blood v1 workflow, which was designed to analyze nucleic acids associated with intact microorganisms. The source workflow used Nextera XT libraries and 1 × 150-bp single-end sequencing on Illumina NextSeq 500/550 instruments; the public run metadata additionally contain NextSeq 550 and MiSeq labels for technical runs. The NTC consisted of DEPC-treated sterile DNase/RNase-free water and was subjected to the same sample-processing, amplification, library-construction, sequencing and bioinformatic workflow as clinical specimens. Human reads were removed before deposition in SRA; consequently, microbial-to-host read ratios could not be reconstructed from the public files and were not inferred.

### 2.3 Read preprocessing, MAG recovery, and quality assessment

Reads were trimmed with fastp v0.23.4 (Chen et al., 2018). Assembly and binning used MetaWRAP (Uritskiy et al., 2018) with MEGAHIT (Li et al., 2016), MetaBAT2 (Kang et al., 2019), MaxBin2 (Wu et al., 2015) and CONCOCT (Alneberg et al., 2014). CheckM2 (Chklovski et al., 2023) evaluated 1,013 bins, of which 13 met the operational thresholds of completeness ≥50% and contamination <10%. Dereplication with dRep (Olm et al., 2017) at a secondary ANI threshold of 0.95 yielded 11 representatives; SRR12994718_bin_011 and SRR12994740_bin_004 were non-representatives. The previously reported completeness − 5 × contamination ≥60 rule was incorrect and was removed. The 11 representatives had completeness values ranging from 50.86 to 68.29% (median, 55.73%) and contamination values ranging from 3.49 to 9.29% (median, 7.06%; Supplementary Table S3). All 11 MAGs were classified as medium-quality drafts. None met the completeness criterion for high-quality MAGs because completeness ranged from 50.86 to 68.29%. Complete rRNA and tRNA inventories were also unavailable. Strain-heterogeneity values were unavailable in the retained CheckM2 outputs and were not assessed. Nine representatives originated from NTC-labeled assemblies; assembly source was therefore reported explicitly, and patient abundance evidence was interpreted only as above-background read support, not as proof of bloodstream origin or recovery of an intact genome in blood.

### 2.4 Read-level taxonomic profiling and viral sequence identification

Read-level taxonomic assignments were generated with Kraken2 (Wood et al., 2019) and summarized in the archived species.tab matrices. These matrices, rather than GTDB-Tk (Chaumeil et al., 2019) or the assembled-contig workflow, were the source of the database label Torque teno virus 22. Viral and non-viral labels were evaluated using the same control-aware framework. The stronger taxon evidence tier required abundance greater than threefold the matched-NTC background, abundance above the feature-specific 95th-percentile NTC envelope, and at least 10 assigned reads. Host and MS2 control labels were excluded from biological interpretation. The exact Kraken2 software version, reference-database build and build date were not retained in the archived analysis materials; taxon names are therefore reported exactly as present in the archived matrix, and species-level confirmation, viability and clinical attribution are not inferred from nomenclature alone.

geNomad (Camargo et al., 2024) was used separately to screen assembled sequences for viral contigs and to provide assembly-level viral annotation, whereas GTDB-Tk was restricted to taxonomic assignment of bacterial and archaeal MAGs. The archived project did not retain the geNomad version, database release, command log, virus_summary.tsv, viral-contig FASTA or a contig-to-Torque teno virus 22 mapping. Accordingly, the read-level Kraken2 label was not treated as independently confirmed by a geNomad contig, and no recovery of a complete or near-complete Torque teno virus genome was claimed. This separation preserves the assembled viral-contig analysis while preventing an unsupported transfer of taxonomic provenance between the Kraken2 and geNomad workflows.

### 2.5 Mobile genetic elements annotation and classification

Predicted ORFs from MAGs were searched against mobileOG-db (Brown et al., 2022) using DIAMOND v2.0.15 in blastp mode with the parameters --evalue 1e-20, −-max-target-seqs 1, −-id 90, −-subject-cover 90 and -f 6. MGE-associated annotations were summarized using the five classes represented in the retained analysis tables: bacteriophages, plasmids, insertion sequences, integrative elements and multiple-class assignments. These categories describe sequence homology and were not interpreted as evidence of excision, replication, integration or transfer activity.

### 2.6 Identification of VFGs, ARGs, and PHI

ORFs from MAGs, phages and plasmids were searched against the Virulence Factor Database (VFDB) (Liu et al., 2022) using DIAMOND v2.0.15 in blastp mode with --evalue 1e-5, −-max-target-seqs 1, −-id 80, −-subject-cover 70 and -f 6. ARG homologs were annotated against SARG v3.2.1 (Yin et al., 2018, 2023) using the same alignment thresholds, and read-level ARG types and subtypes were quantified with ARGs-OAP v3.2.4. To annotate homologs associated with experimentally characterized pathogen–host interaction phenotypes, ORFs were searched against PHI-base at E ≤ 1e-5 (Urban et al., 2024). All VF, ARG and PHI results were treated as sequence-homology annotations rather than demonstrated expression or phenotype.

### 2.7 Statistical analysis and figure generation

Run-level metadata included accession, biological sample identifier, subject label, timepoint, sample role and sequencing batch. For each feature and patient run, the maximum abundance among NTCs from the same batch defined the matched background. Continuous background-excess abundance, max(patient − matched NTC, 0), was the primary quantitative representation. The stronger sensitivity tier required patient abundance >3 × the matched NTC and above the feature-specific 95th percentile across all NTCs; taxonomic calls additionally required ≥10 assigned reads. The >1×, >5 × and >10 × thresholds were retained as supplementary sensitivity analyses. The four duplicate patient–timepoint identifiers were treated as technical replicates and were not counted as independent biological samples; run-level control correction preceded aggregation at the Sample_ID level. NTCs were used for background calibration and were not treated as a clinical comparison group.

Genome-resolved metabolic reconstruction was summarized from the KEGGModuleHit sheet of the METABOLIC output. Modules annotated as present were counted across MAGs to calculate module prevalence, category-level burden, and per-genome module burden. Because these data were MAG-level presence/absence reconstructions rather than sample-level abundance profiles, this analysis was interpreted as a description of genome-resolved metabolic potential rather than longitudinal functional fluctuation.

Read-level ARG type and subtype TPM matrices were processed using the same matched-control framework. Bray-Curtis dissimilarities were calculated from sample-level relative profiles after run-level background correction and aggregation to biological Sample_ID. Distances from post-fever samples to subject-specific pre-fever baselines were calculated only when the required timepoints were available. Two-sided paired Wilcoxon signed-rank tests were performed as descriptive sensitivity analyses when sufficient complete pairs remained. Exact analyzable sample sizes and corresponding *p* values for the continuous and threshold-based comparisons are provided in Supplementary Table S6. Given the small number of complete paired subjects, particularly three for the MAG-derived species comparison and seven for the ARG-type comparison under continuous correction, these longitudinal comparisons were interpreted descriptively and were not used to support claims of temporal equivalence or stability.

Genome-resolved ARG architecture was summarized from MAG-level type, subtype, mechanism-group, and mechanism-subgroup count tables. For each level, prevalence across MAGs, total counts across MAGs, and per-genome burden were calculated. Virulence-factor analyses were performed similarly using MAG-level VF summary tables, from which VF category prevalence, cumulative VF burden, and per-genome VF richness and burden were derived. Mobile genetic element (MGE) analyses were based on MAG-level MGE summary tables, and MGE class prevalence, cumulative burden, and per-genome burden were calculated across recovered genomes.

PHI-associated phenotype analyses were performed using MAG-level PHI summary tables. Because PHI raw annotation classes are highly granular and overlapping, broader phenotype groups were generated by keyword-based collapsing of raw PHI terms into categories such as reduced virulence, unaffected pathogenicity, hypervirulence, loss of pathogenicity, lethal, effector/avirulence, and chemistry target. These broad PHI groups were treated as non-exclusive annotation groups, not as mutually exclusive biological outcome classes.

SRP016572 (Gyarmati et al., 2016) was examined as an external archived dataset with unresolved sample identities. Although the source publication reported 27 blood samples from nine patients, all 34 public WGS runs shared a single experiment and BioSample and were labeled as NTC in the archive. Putative clinical, spike-in and blank roles could therefore only be inferred from sequence-profile characteristics. Because this role inference depended on the same sequence profiles used for downstream analyses, SRP016572 was not used for independent cohort-level validation or patient-level inference. Profile-based exploratory results are shown in Supplementary Figure S7 solely to illustrate the metadata limitation.

All data processing and visualization were performed in R using custom scripts based primarily on tidyverse for data wrangling, vegan for Bray–Curtis dissimilarity and ordination, readxl for parsing METABOLIC Excel outputs, and ggplot2, patchwork, ggrepel, and scales for figure generation.

## 3 Results

### 3.1 Control-aware taxonomic and MAG-derived profiles retained threshold-dependent patient signals

The source study enrolled 20 patients and reported 47 biological blood samples. In the public deposit, 19 patient labels and 43 unique patient–timepoint identifiers were represented by 47 patient runs (10 T1 runs, 21 T2 runs and 16 T3 runs); four sample identifiers had duplicate technical runs. The deposit also contained 23 NTC runs spanning all 21 sequencing batches, and every patient run had a same-batch NTC comparator. Host and MS2 control reads were excluded from the microbial denominator. At read level, continuous matched-NTC subtraction retained 51.63% of unadjusted taxonomic CPM. The >1×, >3×, >5 × and >10 × sensitivity scenarios retained 74.73, 46.12, 38.80 and 31.38%, respectively, demonstrating graded sensitivity to background correction rather than an all-or-none loss of signal.

The taxonomic evidence-tier analysis identified 3,501 taxa with at least one patient occurrence that exceeded both threefold matched-NTC abundance and the global NTC 95th-percentile envelope. Of these, 1,551 taxa met this combined criterion in at least two patient samples, accounting for 6,374 recurrent or singleton stronger-tier above-background sample-level occurrences. These counts were used to describe the breadth and recurrence of above-background taxonomic signals. They were not interpreted as 3,501 independently confirmed bloodstream organisms because broad reference assignments, DNA persistence and unmeasured pre-analytical sources could not be resolved from the public data.

For genome-resolved analysis, 13 candidate bins were dereplicated to 11 representatives with dRep at 0.95 ANI, and all 11 were retained as the reference MAG catalogue. Ten MAGs showed positive continuous background-excess abundance in at least one patient sample, whereas SRR12994692_bin_001 remained background-like. The continuous correction retained 9.18% of the unadjusted MAG-level TPM. Thus, the catalogue preserved genome diversity for annotation, while abundance-based evidence tiers separately indicated how strongly each representative was supported in patient libraries.

The 11 dereplicated MAGs had completeness values ranging from 50.86 to 68.29% (median, 55.73%) and contamination values ranging from 3.49 to 9.29% (median, 7.06%). All 11 MAGs were classified as medium-quality drafts. None met the completeness criterion for high-quality MAGs because completeness ranged from 50.86 to 68.29%. Complete rRNA and tRNA inventories and strain-heterogeneity values were unavailable (Supplementary Table S3).

Above-background patient read support varied markedly among the 11 MAGs. SRR12994727_bin_008, assembled from a patient library, exceeded its matched NTC in 32 samples, exceeded threefold background in 27 and exceeded tenfold background in 10; 15 samples also cleared the global NTC envelope. SRR12994733_bin_011, SRR12994700_bin_006 and SRR12994706_bin_005 had 25, 17 and 18 positive background-excess occurrences, respectively, and each showed recurrent control-envelope support. Together, these four MAGs contributed 24 sample-level occurrences meeting the combined >3x and global-envelope criterion.

Above-background read support was not confined to references assembled from patient-labeled libraries. Nine of the 11 representatives originated from NTC-labeled assemblies and were therefore regarded as probable laboratory-background reference genomes unless patient reads provided additional quantitative support. Several nevertheless had TPM values above their batch-matched NTC in patient libraries. SRR12994729_bin_008 exceeded threefold background in five samples and cleared the global envelope once, whereas SRR12994709_bin_011 exceeded threefold background in three samples without clearing the envelope. Conversely, SRR12994688_bin_024 had a sizeable continuous excess signal but no >3 × occurrence. Because the archived abundance matrix did not retain genome-wide coverage breadth or unique-mapping diagnostics, these patterns were interpreted as above-background read support for reference sequences, not as proof that complete genomes or viable organisms were present in blood.

**Figure 1:**
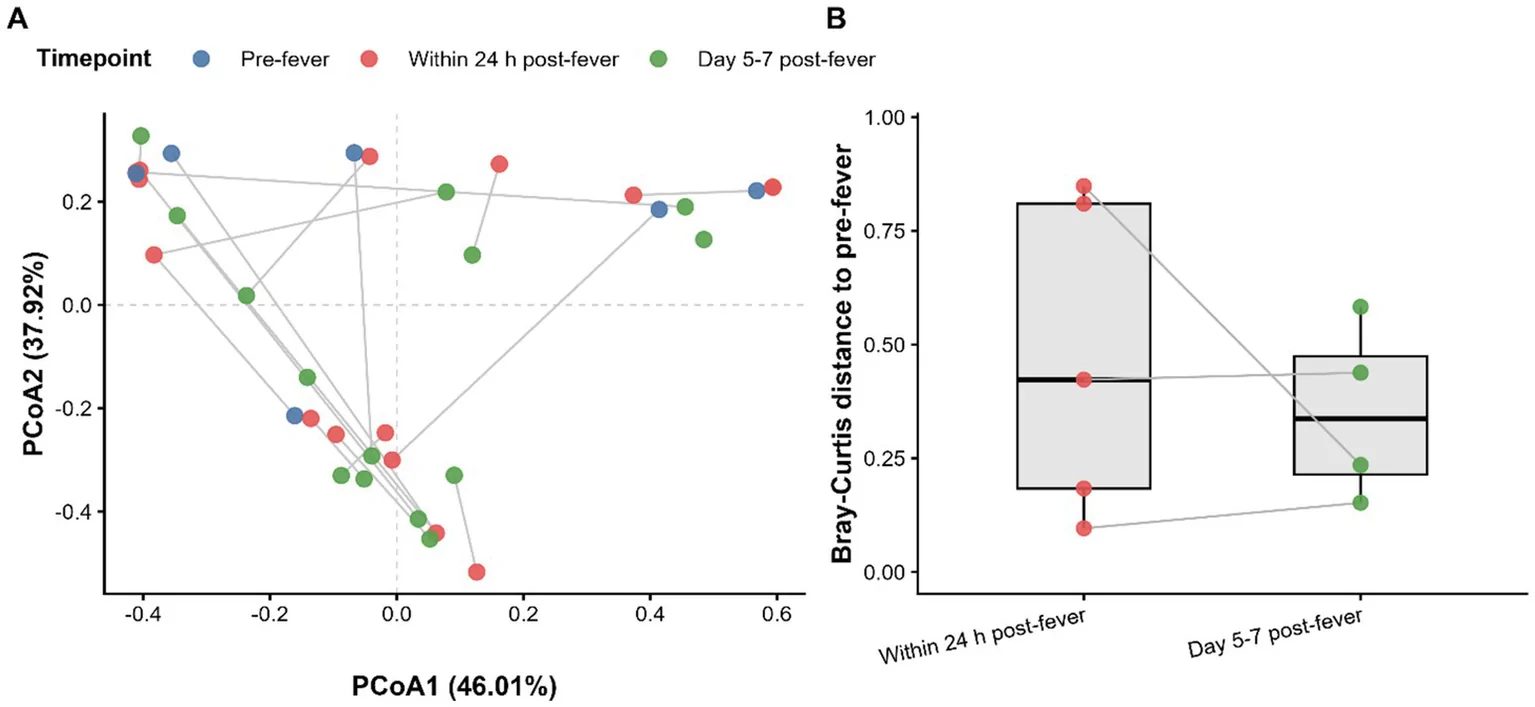
Control-aware longitudinal profiles of MAG-derived species labels. **(A)** Principal coordinate analysis of Bray-Curtis dissimilarities calculated from relative compositions in the continuous background-excess matrix. Eight species-level labels were detected across 35 nonzero biological samples. Blue, red, and green points represent pre-fever, within-24-h post-fever, and day-5-to-7 post-fever samples, respectively; grey paths connect longitudinal samples from the same subject. PCoA1 and PCoA2 explain 46.01 and 37.92% of the variation represented by positive eigenvalues, respectively. **(B)** Bray-Curtis distances between each post-fever sample and its subject-specific pre-fever baseline. Points represent biological samples (within 24 h, *n* = 5; day 5 to 7, *n* = 4), and grey lines connect the three subjects with both post-fever contrasts available. Boxes indicate medians and interquartile ranges, with whiskers extending to 1.5 times the interquartile range. No *p* value is displayed because only three subjects contributed both contrasts; descriptive paired-test results are provided in Supplementary Table S6. Duplicate sequencing runs assigned to the same biological Sample_ID were not treated as independent observations.

The primary MAG-derived species matrix contained eight species-level labels across 35 nonzero biological samples. Bray-Curtis PCoA separated 46.01 and 37.92% of positive-eigenvalue variation on the first two axes (Figure 1A). Thresholding progressively reduced the analyzable matrix: the >3x, >5x and >10x scenarios retained seven, six and three labels across 27, 18 and 10 samples, respectively. This threshold dependence was reported explicitly because increasingly strict background rules improved specificity but also removed quantitative structure needed to assess longitudinal ecological variation.

Distances from each subject’s pre-fever baseline were available for five within-24-h samples and four day-5-to-7 samples (Figure 1B). Only three subjects contributed both post-baseline contrasts under continuous correction; three remained at >1×, two at >3×, and none at >5 × or >10×. Given these small and progressively diminishing paired denominators, the distributions were interpreted descriptively. Exact analyzable sample sizes and paired-test *p* values are reported in Supplementary Table S6. These sparse complete pairs do not support inference of temporal equivalence or stability and instead indicate that genome-resolved longitudinal analysis was substantially constrained after control calibration.

### 3.2 KEGG reconstruction revealed a sparse catalogue with uneven above-background patient read support

**Figure 2:**
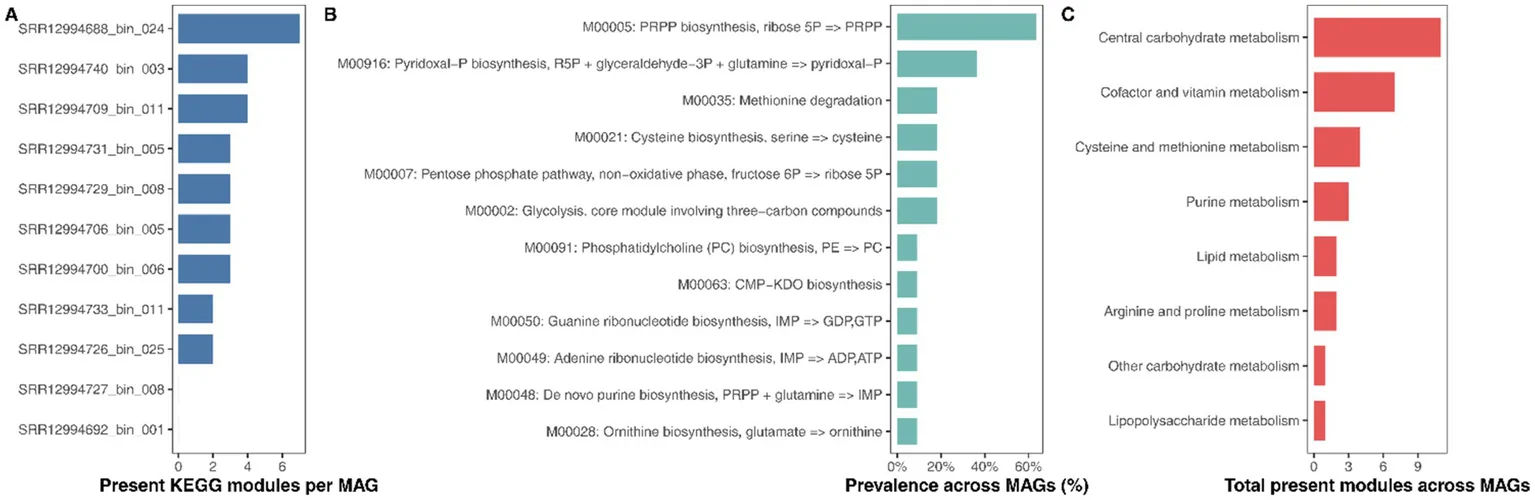
Positive KEGG-module calls across the reference MAG catalogue. **(A)** Number of complete KEGG modules called as present in each of the 11 reference MAGs. **(B)** Prevalence of the 12 most frequently detected modules across the 11 MAGs; bars show the percentage of MAGs with a positive module call. **(C)** Total positive module calls grouped by KEGG module category across the catalogue. The catalogue contained 31 positive module calls, including eight calls in the four recurrent control-envelope-supported MAGs. Bars are descriptive counts or proportions and do not show uncertainty intervals. All MAGs were medium-quality drafts; therefore, unobserved modules were not interpreted as biological absence or pathway inactivity.

The complete 11-MAG reference catalogue contained 31 positive complete-module calls (Figure 2A). Evidence-tier restriction reduced this total in a graded manner: the eight MAGs with at least one >3x patient occurrence contained 20 calls, the seven with recurrent >3x support contained 17, the five with any global-envelope-supported occurrence contained 11, and the four with recurrent global-envelope support contained eight. The three MAGs that reached >10x matched-NTC abundance contained five calls. These nested totals linked functional annotation to abundance support without treating unsupported catalogue members as nonexistent.

Module burden was uneven across genomes, ranging from zero to seven positive calls per MAG. SRR12994688_bin_024 carried seven calls despite lacking a > 3x patient occurrence, whereas SRR12994727_bin_008 generated the strongest and most recurrent patient abundance signal but had no complete KEGG module call. SRR12994692_bin_001 also had no positive module call and no patient background-excess abundance. This discordance showed that assembly completeness, genome content and patient abundance were distinct properties; functional richness could not be inferred from evidence tier, and evidence tier could not be inferred from module count.

PRPP biosynthesis was the most prevalent module, occurring in seven of 11 MAGs (63.64%; Figure 2B). Pyridoxal-phosphate biosynthesis occurred in four MAGs (36.36%). Core three-carbon glycolysis, the non-oxidative pentose phosphate pathway, cysteine biosynthesis and methionine degradation each occurred in two MAGs (18.18%). All other positive modules were singletons. The distribution therefore consisted of a small shared biosynthetic core accompanied by many low-prevalence calls, rather than a uniformly reconstructed metabolic program across the reference catalogue.

At category level, central carbohydrate metabolism contributed 11 positive calls and was represented in eight MAGs (Figure 2C). Cofactor and vitamin metabolism contributed seven calls across six MAGs, while cysteine and methionine metabolism contributed four calls across four. Purine metabolism contributed three calls within one MAG; arginine and proline metabolism and lipid metabolism each contributed two calls across two MAGs. Lipopolysaccharide biosynthesis and the remaining carbohydrate category were represented by one call each, further illustrating the sparse and heterogeneous reconstruction.

These results describe positive module reconstruction in medium-quality draft MAGs and should be read asymmetrically. A complete module call supports the presence of the annotated enzymatic complement within the reconstructed genome, but an unobserved module may reflect incomplete recovery or annotation limits. Consequently, zero calls were not interpreted as biological absence, metabolic inactivity or adaptation to blood. Likewise, modules in a reference MAG were not attributed to a patient unless that MAG also had abundance support in the corresponding patient library; the evidence-set totals provide that required overlay.

### 3.3 Matched-control read-level resistomes retained recurrent but threshold-sensitive patient signals

Continuous matched-NTC correction retained all 19 patient-detected ARG types and 44.87% of unadjusted type-level TPM. The >1x, >3x, >5x and >10x scenarios retained 19, 18, 17 and 15 types, respectively, while retaining 73.77, 32.33, 25.95 and 19.63% of the original TPM. Sample-level occurrence counts declined from 185 under continuous correction to 116, 93 and 75 at >3x, >5x and >10x. The reduction in abundance and occurrence with increasing stringency quantified control sensitivity without converting the resistome into a binary present-or-absent result.

Sixteen of the 19 ARG types exceeded both threefold matched-NTC abundance and the global NTC envelope in at least one patient sample, and nine did so recurrently in two or more samples, yielding 50 stronger-tier above-background sample-level occurrences. Polymyxin-, mupirocin- and rifamycin-associated signals met the combined criterion in nine, eight and six patient samples, respectively. Beta-lactam-associated signals met it in five samples, while aminoglycoside and macrolide-lincosamide-streptogramin signals each met it in three. Multidrug and quinolone categories did not clear the combined criterion despite being detected in the unthresholded patient profiles.

Subtype-level analysis retained substantially more sequence diversity. Continuous correction retained all 232 patient-detected subtypes and 66.11% of unadjusted subtype TPM. Although the >3x, >5x and >10x scenarios retained 232, 231 and 230 subtypes, their retained TPM fractions decreased to 60.67, 54.67 and 51.20%, and the corresponding sample-level occurrence counts decreased from 679 to 646 and 604. Thus, most subtype labels persisted somewhere in the cohort even under strict ratios, but their quantitative contribution and distribution across patients were progressively reduced.

The combined >3x and global-envelope criterion was met by 215 subtypes in at least one patient sample and by 89 subtypes in two or more samples, producing 428 stronger-tier above-background subtype occurrences. Representative recurrent signals included erm(F), ugd and OprM, each supported in eight patient samples; mdtF and acrF were supported in seven; and arnA, abcA, mdtK, erm(X), qacH, aadS and LpeB were each supported in six. These labels were retained as homology assignments at the reported annotation resolution and were not collapsed into claims of phenotypically equivalent resistance.

**Figure 3:**
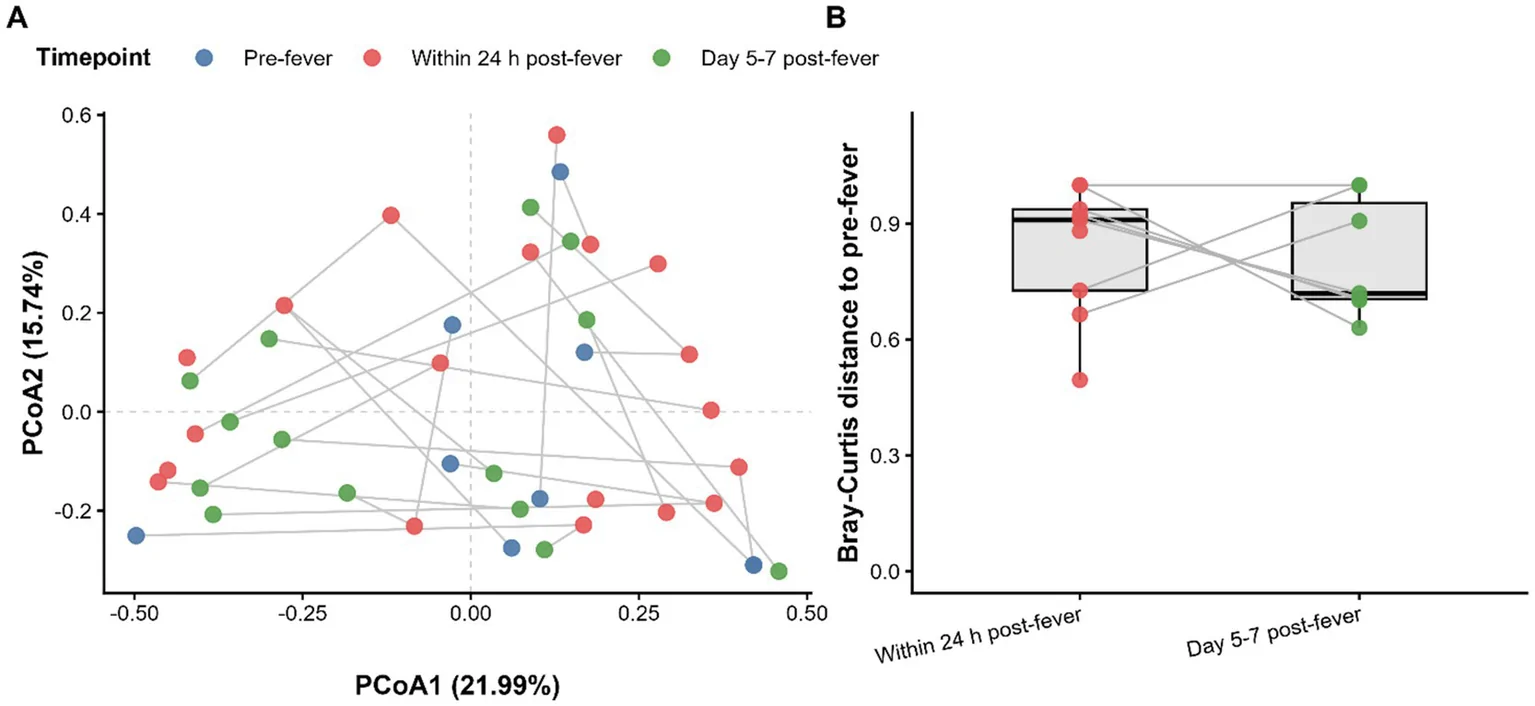
Longitudinal read-level ARG-type profiles after matched-control correction. **(A)** Principal coordinate analysis of Bray-Curtis dissimilarities calculated from relative ARG-type compositions in the continuous background-excess matrix. Nineteen ARG types were detected across 42 nonzero biological samples. Colors represent pre-fever (blue), within-24-h post-fever (red) and day-5-to-7 post-fever (green) samples; grey paths connect longitudinal samples from the same subject. PCoA1 and PCoA2 explain 21.99 and 15.74% of the variation represented by positive eigenvalues, respectively. **(B)** Bray-Curtis distances between each post-fever sample and its subject-specific pre-fever baseline. Points represent biological samples (within 24 h, *n* = 9*; day 5 to 7, n* = 7), and grey lines connect the seven subjects with both post-fever contrasts available. Boxes indicate medians and interquartile ranges, with whiskers extending to 1.5 times the interquartile range. No *p* value is displayed because only seven subjects contributed complete paired contrasts; descriptive paired-test results are provided in Supplementary Table S6.

The continuous ARG-type matrix contained 19 types across 42 nonzero biological samples (Figure 3A), with PCoA1 and PCoA2 explaining 21.99 and 15.74% of positive-eigenvalue variation, respectively. Nine within-24-h and seven day-5-to-7 distances were calculated relative to subject-specific pre-fever baselines (Figure 3B). Seven subjects contributed both post-baseline contrasts under continuous correction and the >1 × threshold; the number of complete pairs decreased to five at >3 × and >5 × and to four at >10×. Given these small and threshold-dependent paired denominators, the longitudinal distributions were interpreted descriptively. Exact analyzable sample sizes and paired-test *p* values are reported in Supplementary Table S6.

Subtype-level paired comparisons included seven complete subjects under the continuous, >1×, >3×, >5 × and >10 × scenarios, with corresponding descriptive test results provided in Supplementary Table S6. These comparisons were limited by small paired sample sizes and did not support inference of temporal equivalence or stability in resistome composition. In addition, read-level ARG homology does not establish transcription or antimicrobial susceptibility phenotype, and the available antibiotic-exposure metadata were insufficient for treatment-response modeling. The results therefore support the presence of recurrent patient-enriched ARG DNA signals, while their temporal and clinical significance remains uncertain.

### 3.4 MAG-associated ARG architectures varied across catalogue and patient read-support tiers

The complete 11-MAG reference catalogue contained 174 ARG homolog hits. The eight MAGs with at least one >3x patient occurrence contained 137 hits, and the seven with recurrent >3x support contained 115. Restriction to the five MAGs with any global-envelope-supported patient occurrence retained 73 hits, whereas the four with recurrent global-envelope support retained 44. The three MAGs reaching >10x matched-NTC abundance contained 30 hits. These nested evidence sets preserved catalogue-level functional diversity while identifying which annotations were linked to increasingly strong patient abundance support.

**Figure 4:**
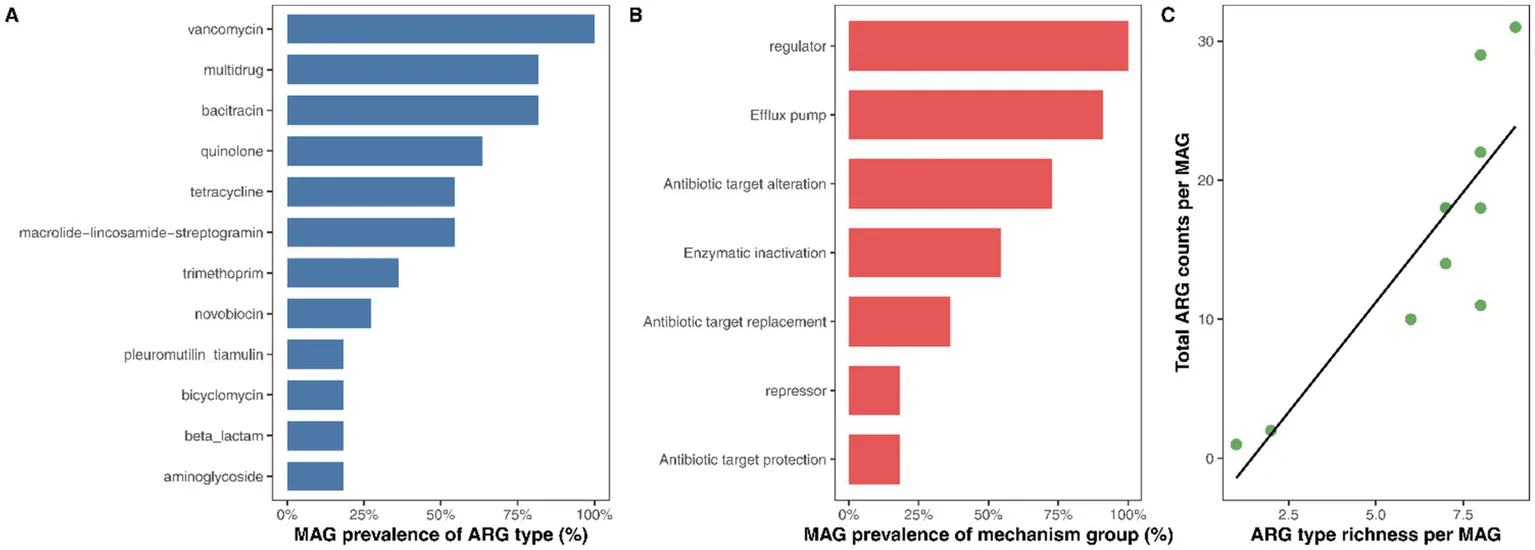
ARG-homology architecture of the 11 reference MAGs. **(A)** Prevalence of the 12 most frequently detected ARG types across the catalogue. Bars show the percentage of MAGs containing at least one homolog assigned to each type. **(B)** Prevalence of ARG mechanism groups across the same 11 MAGs. **(C)** Relationship between ARG-type richness and total ARG-hit burden. Each green point represents one MAG; the black line is an ordinary least-squares fit shown without a confidence interval for descriptive visualization only. The catalogue contained 174 ARG hits; the four recurrent control-envelope-supported MAGs contained 44. No inferential test was applied to panel **(C)**. Annotations indicate sequence homology and do not establish gene expression or resistance phenotype.

Per-MAG burden ranged from one to 31 ARG hits and from one to nine ARG types (Figure 4C). SRR12994709_bin_011 and SRR12994729_bin_008 carried 31 and 29 hits, respectively, but only the latter had one global-envelope-supported occurrence. SRR12994706_bin_005 and SRR12994700_bin_006 carried 18 and 14 hits and showed recurrent global-envelope support, whereas the strongly above-background in patient libraries SRR12994727_bin_008 carried only two hits. Together with SRR12994733_bin_011, these MAGs with recurrent above-background patient read support accounted for the 44-hit core linked to the strongest patient evidence tier.

Vancomycin-associated homologs were detected in all 11 MAGs and contributed 40 catalogue hits (Figure 4A). Multidrug and bacitracin categories each occurred in nine MAGs, contributing 47 and 20 hits, respectively. Quinolone-associated homologs occurred in seven MAGs, while tetracycline and macrolide-lincosamide-streptogramin homologs each occurred in six. Less prevalent categories, including trimethoprim, novobiocin and beta-lactam, occurred in four, three and two MAGs. Prevalence and hit count therefore captured different aspects of ARG architecture.

Mechanism annotations were similarly widespread but heterogeneous (Figure 4B). Regulatory homologs occurred in all 11 MAGs, efflux-related homologs in ten, target-alteration homologs in eight and enzymatic-inactivation homologs in six. Target replacement occurred in four MAGs, while target protection, repressor and other low-prevalence mechanisms were confined to two or fewer. The high prevalence of broad regulatory and efflux categories partly explained why catalogue hit counts exceeded the number of narrowly specified drug-class assignments, and why raw hit burden alone was not used as a clinical resistance score.

MAG association added genomic context to the read-level resistome but did not establish that an ARG homolog was expressed, phenotypically active or carried by a viable circulating organism. The fitted relation between ARG-type richness and total hits in Figure 4C was descriptive only. For NTC-assembled reference MAGs, patient TPM above the matched-control background was reported as read support rather than as evidence of bloodstream origin; confirmation would require genome-wide coverage breadth, unique mapping, targeted assays or isolate data. Phenotypic resistance would additionally require susceptibility or expression measurements, which were unavailable.

### 3.5 Virulence-factor homologs were heterogeneous and concentrated in MAG subsets with above-background patient read support

The full 11-MAG catalogue contained 402 virulence-factor (VF) homolog hits. The eight MAGs with at least one >3x patient occurrence contained 328 hits, and the seven with recurrent >3x support contained 267. Five MAGs with any global-envelope-supported occurrence contained 167 hits, while the four with recurrent support contained 127. The three MAGs reaching >10x matched-NTC abundance contained 91 hits. As for other annotation classes, these nested totals distinguished the complete reference inventory from the subset most consistently linked to patient abundance.

**Figure 5:**
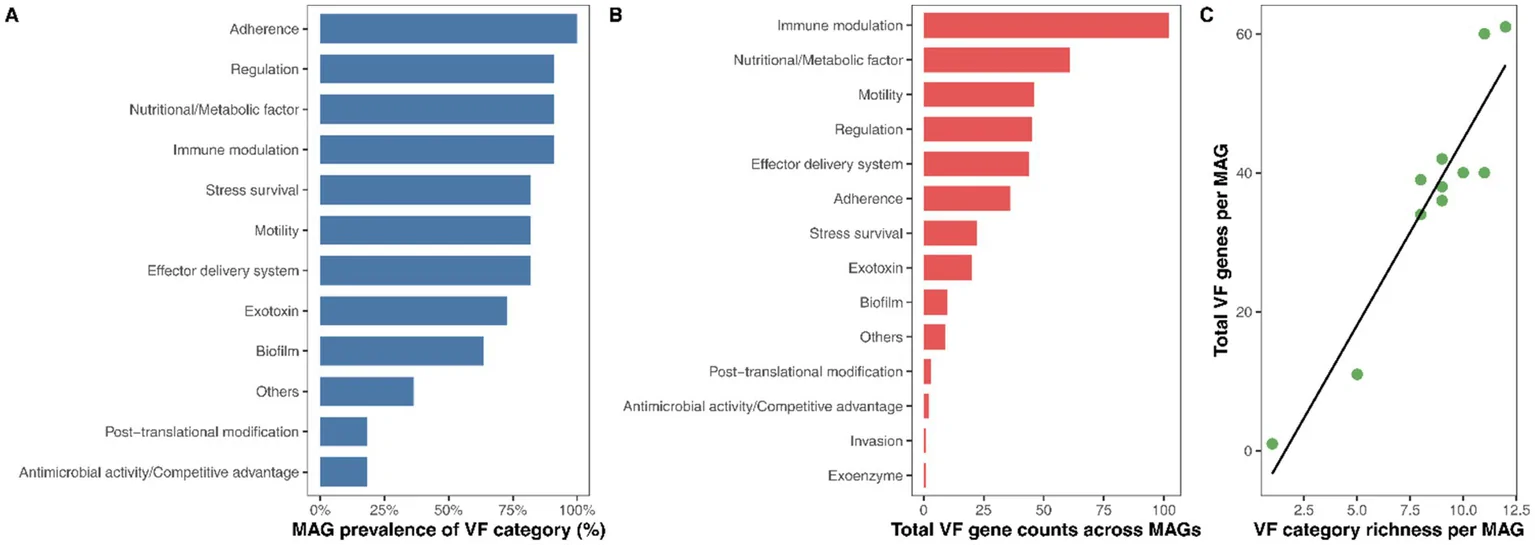
Virulence-factor homology across the 11 reference MAGs. **(A)** Prevalence of the 12 most frequently represented VF categories. Bars show the percentage of MAGs containing at least one homolog in each category. **(B)** Total VF-hit counts for all detected categories across the catalogue. **(C)** Relationship between VF-category richness and total VF-hit burden per MAG. Each green point represents one MAG; the black line is an ordinary least-squares fit without a confidence interval and is descriptive only. The full catalogue contained 402 VF hits; the four recurrent control-envelope-supported MAGs contained 127. No inferential test was applied to panel **(C)**. Positive VF homology does not establish expression, invasive capacity or pathogenicity.

Per-MAG VF burden ranged from one to 61 hits, and category richness ranged from one to 12 (Figure 5C). SRR12994731_bin_005 and SRR12994709_bin_011 carried 61 and 60 hits but lacked global-envelope support, whereas recurrently supported SRR12994706_bin_005, SRR12994700_bin_006, SRR12994727_bin_008 and SRR12994733_bin_011 carried 42, 36, 11 and 38 hits, respectively. The resulting 127-hit recurrent set was therefore not simply the four most heavily annotated genomes, again separating functional burden from strength of patient occurrence evidence.

Adherence-associated homologs occurred in all 11 MAGs (Figure 5A). Immune-modulation, nutritional or metabolic, and regulation categories each occurred in ten MAGs. Motility, effector delivery and stress-survival homologs each occurred in nine, exotoxin-associated homologs in eight and biofilm-associated homologs in seven. The breadth of category prevalence indicated that many reference genomes encoded homologs assigned to host-interaction functions, but it did not determine whether the relevant genes were intact, expressed or exposed to host tissues.

Immune-modulation annotations were the largest count category, contributing 102 hits across the catalogue (Figure 5B). Nutritional or metabolic factors contributed 61 hits, motility 46, regulation 45 and effector delivery 44. Adherence contributed 36 hits, stress survival 22, exotoxin 20 and biofilm 10, while other categories were less abundant. The ranking differed from prevalence because some widely distributed categories contained few homologs per MAG, whereas immune-modulation annotations accumulated multiple hits in individual genomes.

VF annotations were therefore retained as positive sequence-homology findings rather than converted into a pathogenicity label. The descriptive line in Figure 5C summarized the relation between category richness and total burden but was not an inferential model. Medium-quality assembly can create false-negative category calls, while database homology can include proteins with context-dependent or incompletely characterized functions. Clinical virulence, invasion and disease causation would require organism-level confirmation, expression or experimental phenotype data, none of which were available in the public blood metagenomic dataset.

### 3.6 MGE and PHI annotations showed distinct distributions across evidence tiers

The complete reference catalogue contained 208 mobile genetic element (MGE) hits. The eight MAGs with at least one >3x patient occurrence contained 127 hits, the seven with recurrent >3x support contained 73, and the five with any global-envelope-supported occurrence contained 34. The four MAGs with recurrent global-envelope support contained 26 hits, whereas the three MAGs reaching >10x matched-NTC abundance contained 14. The stepwise decrease showed that much of the catalogue-level MGE inventory belonged to MAGs with weaker patient occurrence evidence, but it did not invalidate those reference annotations.

**Figure 6:**
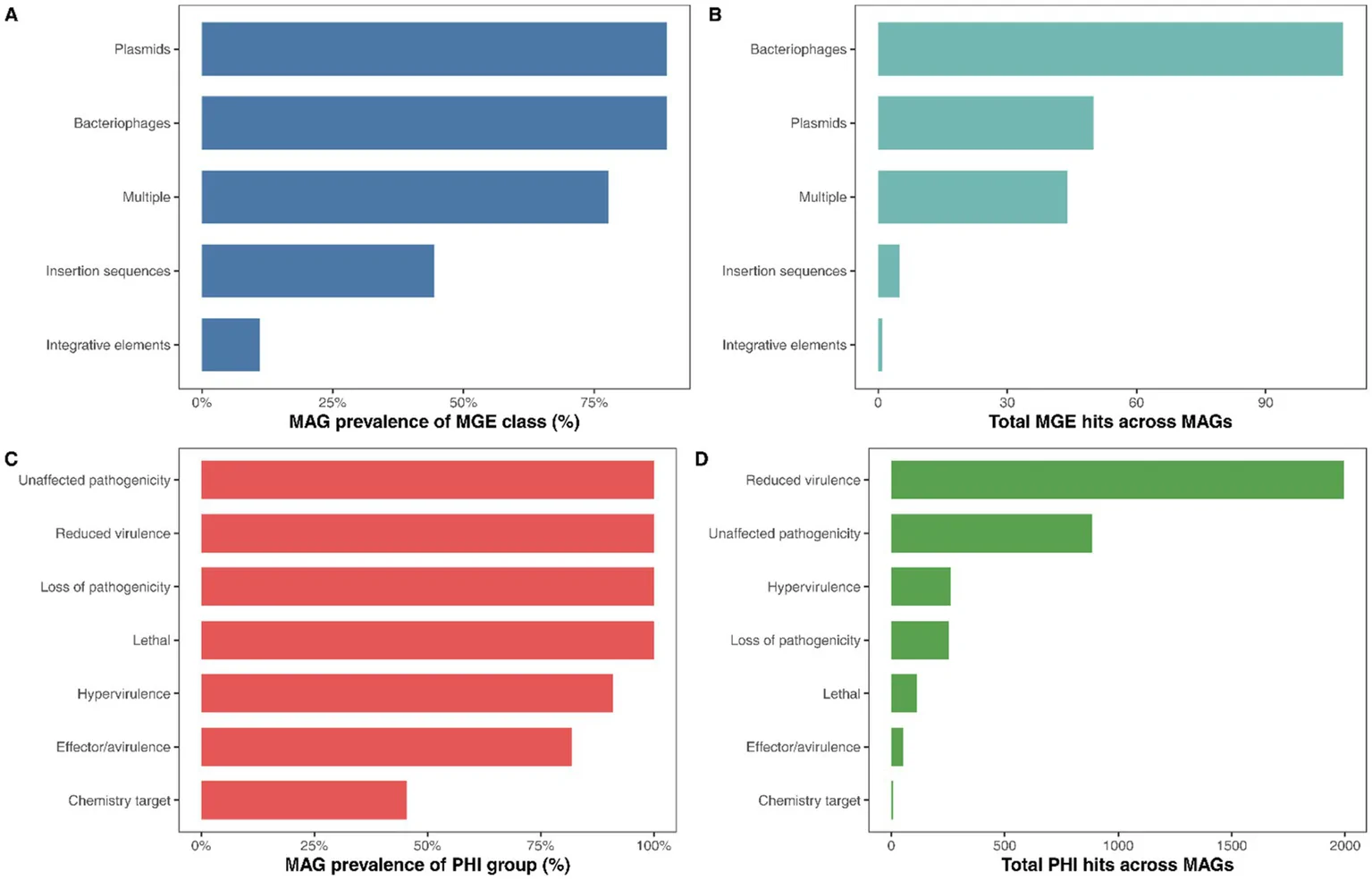
MGE and PHI-base homology summaries across reference MAGs. **(A)** Prevalence of five MGE classes among the nine MAGs represented in the MGE summary table. **(B)** Total MGE-hit counts across those nine MAGs, grouped as bacteriophages, plasmids, multiple-class annotations, insertion sequences and integrative elements. **(C)** Prevalence of seven keyword-collapsed PHI annotation groups across all 11 reference MAGs. **(D)** Total PHI annotation assignments in each group. PHI groups are non-exclusive because a raw term can match more than one keyword-defined category; group totals therefore do not equal the catalogue-level total of 3,054 PHI hits. The four recurrent control-envelope-supported MAGs contained 26 MGE and 1,128 PHI hits. Bars are descriptive and no inferential tests were applied. MGE homology does not demonstrate active transfer, and PHI annotation does not demonstrate an observed host-interaction phenotype.

Nine MAGs were represented in the MGE summary table. Bacteriophage and plasmid annotations each occurred in eight of these nine genomes, multiple-class annotations in seven, insertion sequences in four and integrative elements in one (Figure 6A). Bacteriophage homologs contributed 108 hits, plasmids 50 and multiple-class assignments 44; insertion sequences and integrative elements contributed five and one (Figure 6B). These data documented the distribution of mobile-element homology but did not measure excision, replication, transfer or integration activity.

PHI-base annotation was considerably denser, with 3,054 hits across all 11 MAGs. The eight MAGs with at least one >3x patient occurrence contained 2,548 hits, the seven with recurrent >3x support contained 2,195, and the five with any global-envelope support contained 1,451. The four MAGs with recurrent above-background patient read support retained 1,128 hits, while the three >10x MAGs retained 833. Per-MAG PHI burden ranged from 14 hits in SRR12994692_bin_001 to 456 in SRR12994709_bin_011, illustrating substantial genome-level heterogeneity.

Keyword-collapsed reduced-virulence, unaffected-pathogenicity, loss-of-pathogenicity and lethal groups were represented in all 11 MAGs (Figure 6C). Across the catalogue, these groups contained 1,994, 886, 254 and 111 assignments, respectively (Figure 6D). Hypervirulence terms contributed 260 assignments and occurred in ten MAGs; effector or avirulence terms contributed 53 and occurred in nine. Six chemistry-related assignments occurred in five MAGs. The numerical dominance of reduced-virulence terms reflected database annotation composition rather than an observed phenotype in this cohort.

PHI groups were non-exclusive because one raw annotation could match more than one keyword-defined category, so group totals were not expected to equal 3,054 and were not compared as independent events. Similarly, MGE proximity to ARG or VF homologs was not evaluated here and no horizontal-transfer claim was made. The supported scientific result was that reference MAGs carried diverse MGE and PHI homologs, including in MAGs with recurrent above-background patient read support; activity, transfer direction, host interaction and clinical consequence remained unresolved by sequence annotation alone.

### 3.7 Read-depth stratification of Torque teno virus 22 and limits of external comparison

The Torque teno virus 22 result queried by the reviewer originated specifically from the Kraken2 read-level species table, not from GTDB-Tk and not from a contig-level taxonomic assignment. In PRJNA674642, this database label occurred in four patient runs—SRR12994714 (sample 13.1; 34 reads), SRR12994699 (sample 16.2; 1 read), SRR12994723 (sample 7.3; 345 reads) and SRR12994715 (sample 8.2; 1 read)—and in none of the 23 discovery NTC runs. Thus, the complete observed signal was retained, but its strength was stratified: the 34- and 345-read detections met the predefined ≥10-read requirement of the stronger taxon evidence tier, whereas the two single-read assignments were reported as low-depth observations rather than robust positives.

SRP016572 was not treated as an independent validation dataset. All 34 public WGS runs shared a single experiment and BioSample and were labeled as NTC in the archive, such that putative clinical, spike-in and blank roles could only be inferred from sequence-profile characteristics. Because these inferred roles were derived from the same profiles used in downstream analyses, the external analysis was considered circular and was not used for independent biological validation or patient-level inference. Profile-based exploratory results are provided in Supplementary Figure S7.

## 4 Discussion

This reanalysis showed that the strength of blood-associated microbial DNA signals in PRJNA674642 depended substantially on the stringency of negative-control calibration. Negative controls are known to have a substantial influence on apparent microbial signals in low-biomass sequencing datasets (Salter et al., 2014; Eisenhofer et al., 2019). By integrating batch-matched continuous background correction, ratio-based sensitivity thresholds and a feature-specific global NTC envelope, our analysis separated the full reference catalogue from progressively stronger evidence of patient-associated enrichment. This distinction revealed that only a subset of microbial DNA and ARG signals remained supported as control criteria became more stringent, whereas several catalogue-level annotations showed limited or no comparable evidence in patient libraries. The resulting framework therefore provided a more explicit assessment of evidential strength across taxonomic, resistome and genome-resolved layers without equating reference-catalogue membership with biological support in blood samples.

Genome-resolved longitudinal inference was strongly constrained after control calibration. Only three subjects contributed both post-fever contrasts under continuous correction, and the number of complete pairs decreased further as more stringent thresholds were applied. These comparisons were therefore interpreted descriptively and do not support conclusions of temporal equivalence or stability. Interpretation was additionally limited by the provenance of the MAG catalogue, because nine of the 11 representatives originated from NTC-labeled libraries and the archived abundance matrices lacked genome-wide coverage breadth and unique-mapping diagnostics. Although above-background abundance in patient libraries provides additional support for reads related to these reference sequences, it does not demonstrate recovery of intact genomes from blood or establish organism viability. These MAGs are therefore most appropriately regarded as draft reference sequences with varying degrees of above-background patient read support rather than as confirmed circulating genomes.

The functional results are best understood as positive homology annotations within medium-quality draft reference genomes. A complete KEGG module or ARG, VF, MGE or PHI homolog supports a sequence-level call within the reconstructed fraction, whereas an unobserved pathway cannot be interpreted as biological absence. Patient-to-control enrichment provides a separate abundance overlay but does not demonstrate expression, phenotype, viability or clinical causation.

Antimicrobial exposure is a major unresolved confounder. The source study reported that T2 was collected before empiric antibiotics in 14 patients and after treatment initiation in six, but drug-specific prophylactic and empiric regimens were available only as overlapping cohort-level counts rather than patient-resolved covariates. Patient-level drug–ARG associations and treatment-response models could therefore not be performed. Longitudinal patterns are interpreted as fever-phase-associated microbial DNA variation, not as effects of antibiotic selection. The same evidence boundary applies to virulence and mobility: sequence homology does not establish pathogenicity or active horizontal transfer.

Interpretation of SRP016572 was constrained by unresolved public metadata. Although the source publication described a clinically defined blood cohort, all 34 archived WGS runs shared a single BioSample and experiment and were labeled as NTC, preventing independent assignment of patient, timepoint and control identities. Putative run roles could therefore only be inferred from sequence-profile characteristics, creating circularity when the same profiles were subsequently used for comparison. Consequently, SRP016572 was treated as exploratory external context rather than as independent biological validation (Supplementary Figure S7). Future studies should incorporate verified run-to-subject mappings, absolute microbial biomass measurements, complete software and database provenance, genome-wide coverage criteria, orthogonal confirmation by culture or targeted molecular assays, and prospectively collected outcome-linked clinical metadata to determine which blood-associated microbial DNA signals are biologically reproducible and clinically informative.

## 5 Conclusion

This reanalysis provides a run-resolved, nested evidence framework for distinguishing reference-catalogue content from progressively stronger evidence of patient-associated enrichment. Applied to PRJNA674642, this approach identified recurrent microbial DNA and ARG signals that remained detectable across increasingly stringent matched-control criteria, while also revealing substantial threshold dependence and limited capacity for longitudinal inference. The draft MAG catalogue provides sequence-level functional and genomic context but does not establish the presence of viable bloodstream organisms or clinically expressed phenotypes. Overall, the findings support cautious interpretation of patient-associated microbial DNA and ARG signals in low-biomass blood metagenomic data and should be regarded as hypothesis-generating. Prospective studies with verified sample identities, absolute microbial biomass measurements, orthogonal microbiological confirmation and outcome-linked clinical metadata will be required to establish biological reproducibility and evaluate potential clinical relevance.

## Data Availability

The original contributions presented in the study are included in the article/Supplementary material, further inquiries can be directed to the corresponding author.
