# Supplementary material for: Metagenomic profiling of blood-associated microbial DNA signatures in leukemia-associated febrile neutropenia
Source: Front Microbiol. 2026 Aug 28;17:1917043. doi: 10.3389/fmicb.2026.1917043 (PMC13562036; doi:10.3389/fmicb.2026.1917043)
Supplement: Supplementary file 1 [file Supplementary_file_1.DOCX]

Supplementary Material

# 1 Supplementary Figures

**Fig. S1 Supplementary context for recovered species-level signals in blood metagenomes.** **A**, Recovered species landscape across patient samples, showing that the species-level signal was dominated by a limited number of recurrent taxa together with multiple unclassified MAG-derived bins. **B**, Quality overview of MAGs used for species-level profiling. The recovered MAG set was limited in size and generally of moderate quality, supporting cautious downstream ecological interpretation.

**Fig. S2 Supplementary views of KEGG module distribution across recovered blood MAGs. A**, Presence–absence heatmap of the top reconstructed KEGG modules across MAGs. Module reconstruction was highly uneven, with most modules restricted to a subset of genomes rather than being broadly universal. **B**, KEGG module category landscape summarizing total reconstructed module counts, category prevalence across MAGs, and mean module burden per MAG. The reconstructed functional space was dominated by a small number of central metabolic categories and remained sparse overall.

**Fig. S3 Supplementary views of the read-level ARG landscape across patient blood samples. A**, ARG type landscape summarizing prevalence and mean abundance across patient samples. The resistome was dominated by a limited number of recurrent ARG classes, especially multidrug, tetracycline, macrolide-lincosamide-streptogramin, bacitracin, and rifamycin. **B**, Top ARG subtypes ranked by mean abundance across patient samples. The strongest subtype-level signals were driven by determinants such as multidrug_RanA, bacitracin_bacA, tetracycline_tetA(P), and rifamycin_rphB, together with several additional multidrug- and tetracycline-associated features.

**Fig. S4 Fine-scale subtype and mechanism-subgroup structure of genome-resolved ARG repertoires. A**, Top ARG subtypes ranked by prevalence across MAGs. The most recurrent subtype-level features included multidrug-associated determinants, bacitracin-related genes, multiple vancomycin-associated genes, quinolone_arlR, and tetracycline_tetA(48). **B**, Top ARG mechanism subgroups ranked by prevalence across MAGs. The dominant subgroup-level signals were led by MFS and ABC family antibiotic efflux pumps, together with several target- or pathway-associated resistance features such as undecaprenyl pyrophosphate, vanY family, van ligase, and dihydrofolate reductase.

**Fig. S5 Supplementary views of the genome-resolved VF landscape across recovered blood MAGs. A**, VF category landscape integrating MAG prevalence, total VF gene counts, and mean burden per MAG. Categories related to adherence, immune modulation, nutritional/metabolic factor, regulation, motility, and effector delivery system dominated the recovered functional space. **B**, VF categories ranked by mean burden per MAG. Immune modulation showed the highest average burden, followed by nutritional/metabolic factor, motility, regulation, and effector delivery system.

**Fig. S6 Supplementary views of MGE and PHI-associated burden across recovered blood MAGs. A**, Distribution of total MGE hits per MAG, showing that mobilome-associated burden was uneven across genomes, with a limited subset carrying comparatively dense MGE signal. **B**, Relationship between raw PHI class richness per MAG and total PHI hits per MAG. Genomes with larger PHI-associated repertoires also tended to encode broader phenotype-linked annotation sets.

Fig. S7 Exploratory SRP016572 profiles after sequence-profile-based run-role inference. A, Prevalence and mean continuous background-excess CPM for the 15 Kraken2-derived read-level taxonomic labels with the highest mean abundance across 27 profile-inferred clinical-like runs. B, Mean continuous background-excess TPM of read-level ARG types. C, Mean continuous background-excess TPM of the 20 highest-ranking ARG subtypes. Point area is proportional to prevalence. Run roles were inferred from sequence profiles and were not verified by repository metadata; this figure is included solely to illustrate the metadata limitation and does not constitute independent validation or clinical replication.
